# Mycoviruses: Antagonistic Potential, Fungal Pathogenesis, and Their Interaction with *Rhizoctonia solani*

**DOI:** 10.3390/microorganisms11102515

**Published:** 2023-10-09

**Authors:** Muhammad Umer, Mustansar Mubeen, Qaiser Shakeel, Sajjad Ali, Yasir Iftikhar, Rabia Tahir Bajwa, Naureen Anwar, Muhammad Junaid Rao, Yuejun He

**Affiliations:** 1 Forestry College, Research Centre of Forest Ecology, Guizhou University, Guiyang 550025, China; umer@gzu.edu.cn; 2 Institute for Forest Resources & Environment of Guizhou, Guizhou University, Guiyang 550025, China; 3 Department of Plant Pathology, College of Agriculture, University of Sargodha, Sargodha 40100, Pakistan; mustansar01@yahoo.com (M.M.); yasir.iftikhar@uos.edu.pk (Y.I.); 4 Cholistan Institute of Desert Studies, The Islamia University of Bahawalpur, Bahawalpur 63100, Pakistan; qaiser.shakeel@iub.edu.pk (Q.S.); rabia.kpr@gmail.com (R.T.B.); 5 Department of Entomology, Faculty of Agriculture and Environment, The Islamia University of Bahawalpur, Bahawalpur 63100, Pakistan; sajjad.ali@iub.edu.pk; 6 Department of Biology, Virtual University of Pakistan, Lahore 54000, Pakistan; naureen.anwar@vu.edu.pk; 7 State Key Laboratory for Conservation and Utilization of Subtropical Agro-Bioresources, Guangxi Key Laboratory of Sugarcane Biology, College of Agriculture, Guangxi University, Nanning 530004, China

**Keywords:** mycoviruses, rice sheath blight, *Rhizoctonia solani*, mycovirome, characterization

## Abstract

Mycoviruses, or fungal viruses, are prevalent in all significant fungal kingdoms and genera. These low-virulence viruses can be used as biocontrol agents to manage fungal diseases. These viruses are divided into 19 officially recognized families and 1 unclassified genus. Mycoviruses alter sexual reproduction, pigmentation, and development. Spores and fungal hypha spread mycoviruses. Isometric particles mostly encapsulate dsRNA mycoviruses. The widespread plant-pathogenic fungus *Rhizoctonia solani*, which has caused a rice sheath blight, has hosted many viruses with different morphologies. It causes significant crop diseases that adversely affect agriculture and the economy. Rice sheath blight threatens the 40% of the global population that relies on rice for food and nutrition. This article reviews mycovirology research on *Rhizoctonia solani* to demonstrate scientific advances. Mycoviruses control rice sheath blight. Hypovirulence-associated mycoviruses are needed to control *R. solani* since no cultivars are resistant. Mycoviruses are usually cryptic, but they can benefit the host fungus. Phytopathologists may use hypovirulent viruses as biological control agents. New tools are being developed based on host genome studies to overcome the intellectual challenge of comprehending the interactions between viruses and fungi and the practical challenge of influencing these interactions to develop biocontrol agents against *significant* plant pathogens.

## 1. Introduction

The rice plant, a member of the *Poaceae* family, is grown for its seeds and used as a common cereal grain in tropical, subtropical, and mild temperate climates. Almost 40% of the world’s population relies on rice for food and nutrition, making it the second most essential crop after wheat [1]. Species of both wild and domesticated rice make up the genus *Oryza*. Two kinds of rice are cultivated, including *Oryza sativa* (Asian rice) and *Oryza glaberrima* (African rice); however, there are twenty-two species of wild rice [2,3,4]. Many pests and diseases affect the rice crop, significantly hampering efforts to increase rice output and quality [1]. Sheath blight, a crucial disease in rice, was initially identified in Japan [5,6]. It is one of the major diseases of rice caused by *Rhizoctonia solani*, resulting in severe economic and yield losses of up to 25–40% [7]. For instance, in Asia, rice sheath blight caused by the pathogenic *R. solani* alone results in up to 50% production losses [8]. Other diseases associated with sheath blight include aggregate sheath spot, which is brought on by the multinucleate *R. oryzae* (teleomorph *Waitea circinata*), and sheath spot, which is brought on by the binucleate *R. oryzae sativae* (teleomorph *Ceratobasidium oryzae sativae*) [9,10]. Depending on the host plant, *R. solani* infection symptoms include seedling damping, stem canker, and root or stem rot [9,10,11]. Sclerotia that overwinter and sheaths on the leaf are the typical signs of *R. solani* disease. Breeding tactics, chemical fungicides, and crop rotation are alternatives to employing agronomic methods to manage *R. solani* [12]. Quick solutions often involve the extensive application of chemicals, which raises concerns about high costs, environmental safety, and disease resistance [13]. It is necessary to choose sustainable alternatives without (−ve) any influence on the surrounding environment and crop production [12]. Mycoviruses are fungi-infecting viruses [14]. Identifying hypovirulence-associated mycoviruses in additional plant fungal diseases was spurred by the discovery of the effective biological control of chestnut blight using hypovirus-mediated hypovirulence [15]. Mycoviruses with low virulence might have use as biocontrol agents to manage agricultural diseases [14,16].

## 2. Pathogenesis Biology of *Rhizoctonia solani*

French mycologist Augustin Pyramus de Candolle originally characterized the basidiomycete Rhizoctonia in 1815 [17]. *R. solani* is a soil-borne phytopathogenic fungus with a wide range of hosts and a widespread geographic distribution. It is known to cause several essential crop diseases, which negatively impact agriculture and the economy. More than 3.2 million hectares in China have been infected by this fungus, causing yield losses of more than 200 million kg per year [5]. Appearing in natural circumstances, *R. solani* is a semi-saprophytic pathogen that can harm more than 15 families, including maize, soybean, grain crops, horticultural crops, and several other genera [18,19]. Temperature and soil moisture play important roles in disease development. The optimum temperature for this pathogen is 20–25 °C, and 20% moisture is required for its growth [20]. Although the fungus *R. solani* has a thin and weblike basidiocarp, it is encountered in its anamorphic state as sclerotia and hyphae. The teleomorph is *Thanetophorus cucumeris* [21]. The earliest stage of the fungus *R. solani*, which is based on Thanatephorus cucumeris, appears on leaves, soil, and diseased sheaths slightly above the ground as a thin, mildew-like growth [22]. The best method of preventing rice sheath blight is still the use of resistant cultivars [23]. The resistance factor relies on numerous genes as a quantitative characteristic with a large usable resistance QTL (quantitative trait locus) [24]. This specific approach to managing the fungus *R. solani* does not work well when chemical fungicides like carbendazim and triazole are applied [25]. There are several cases of Trichoderma being used to manage sheath blight. According to Chen et al. [25] and de França et al. [26], volatile secondary metabolites and *Trichoderma asperellum* aqueous solution can effectively control rice sheath blight. Regular disease surveillance should begin at the period of panicle differentiation and continue through the heading. Sheath blight infection after heading may result in considerable financial losses [22].

## 3. Mycoviruses

**Figure A1:**
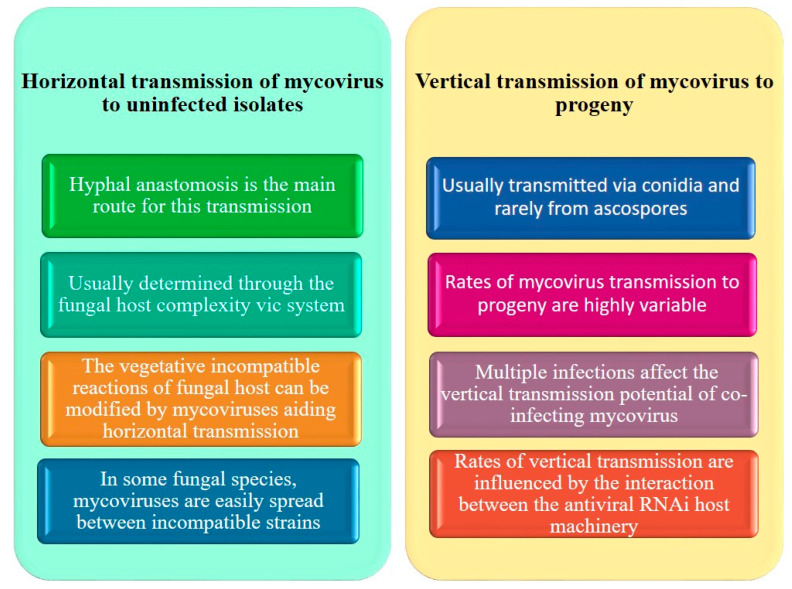
Mycoviruses’ potential to efficiently spread within the pathogen population.

In the edible mushroom *Agaricus bisporus* (phylum: *Basidiomycota*), the first mycoviruses were discovered in 1962 [27]. Since then, the fungal taxa have all been shown to include mycoviruses [28,29]. To find new, unidentified mycoviruses, next-generation sequencing (NGS) techniques are now being applied [30]. Although ssRNA and DNA viruses have been found, the most commonly described are mycovirus dsRNA genomes [29]. According to the International Council for the Taxonomy of Viruses (ICTV), fungal viruses are now grouped into 19 officially recognized families (Table A1) and 1 floating genus that has not yet been assigned to a family. These include the straight dsRNA viruses (*Partitiviridae*, *Amalgaviridae*, *Totiviridae*, *Botybirnavirus*, *Chrysoviridae*, *Quadriviridae*, *Megabirnaviridae*, and *Reoviridae*) and the linear positive-sense (+) ssRNA families (*Alphaflexiviridae*, *Barnaviridae*, *Botourmiaviridae*, and *Endornaviridae* [31,32]. Mycoviruses have the potential to spread within the pathogen population efficiently (Figure A1).

### Symptoms of Mycoviruses and Their Interactions with Hosts

Some significant alterations include altered sexual reproduction, aberrant pigmentation, and uneven development. There are two categories of phenotypic changes: those that are helpful to the host of mycoviruses and those that are destructive. Harmful interactions include hypovirulence and debilitation, which have been found in several plant-pathogenic fungi, such as root rot and rot fungi. Advantageous interactions are identified in fewer plant endophytic and pathogenic fungi, where virulence and heat tolerance increase [33]. The host fungus for mycoviruses is increasingly recognized as a model filamentous fungal organism for virus–virus interaction and viruses’ interactions with their hosts [34].

## 4. Transmission Properties of Fungi

To accurately avoid transmission to unfavorable hosts, the mycovirus must be able to establish and spread inside the selected host population [35]. There are two recognized modes of transmission: vertical transmission via sporulation and transmission through the hypha of fungi and heterokaryons [36], which results in extensive coverage of the biocontrol agent. This kind of transmission is connected to increased biocontrol mechanisms as opposed to vertical transmission, which is often associated with lower efficiency [37]. There have been several instances of horizontal transmission in mutualistic symbioses [38]. *Sclerotinia sclerotiorum* hypovirulence-associated DNA virus-1 (SsHADV-1) is a new circular ssDNA virus that may spread extracellularly and uses the mycophagous insect *Lycoriella ingénue* as a vector for transmission (Figure A1) [39,40].

## 5. Transmission of Viruses in *Rhizoctonia solani*

Successful transfection techniques have previously been used for various mycoviruses, including those from the families *Partitiviridae*, *Megabirnaviridae*, *Totiviridae*, and *Reoviridae*. This method often necessitates using polyethylene glycol (PEG) 4000 [41]. The transmission of RsPV2/GD-11-purified particles, either horizontally or vertically, has been successful [42]. Moreover, members of the Endornaviridae family, which do not create viral particles, can effectively transmit horizontally and vertically. For instance, the *alphaendornavirus* RsEV1/GD-2 can spread horizontally using its hyphal anastomosis, while a *betaendornavirus* identified in *R. solani* Ra1 could spread vertically through basidiospores [11]. Moreover, hyphal anastomosis might transmit the *betapartitiviruses* RsPV6/YNBB-111, RsPV7/YNBB-111, and RsPV8/YNBB-111 horizontally (Figure A1) [43,44].

## 6. Mycoviruses of *Rhizoctonia solani*

The pathogenic fungus *R. solani* contains the first dsRNA element ever reported, being discovered by Butler and Castano [29]. Pathogenic *R. solani* isolates have so far been found to contain about 100 viral infections, consisting of several known families of viruses that contain dsRNA, positive ssRNA, and negative ssRNA, as well as individuals of suggested families and undefined RNA components. On the one hand, certain viruses identified as infecting *R. solani* are members of families of mycoviruses that have been extensively investigated, including the *Barnaviridae*, *Botourmiaviridae*, *Endornaviridae*, *Deltaflexiviridae*, *Hypoviridae*, *Narnaviridae*, *Megabirnaviridae*, and *Partitiviridae*. On the other hand, some are proposed members of the orders *Bunyavirales*, *Tymovirales*, and *Serpentovirales* and belong to or are very similar to families historically recognized as affected plants, such as CMV [11,45]. *R. solani* AG-1 IA, a pathogenic fungus isolated from rice crop, has been found to be infected by several viruses, including *R. solani* dsRNA virus 1 (RsRV1) in 2013 [46], *R. solani* partitivirus 2 (RsPV2) in 2014 [41], and *R. solani* RNA virus 2 (RsRV2) [47]. Also, more recently, *Rhizoctonia solani* partitiviruses 3 to 8 (RsPV3 to 8, respectively), *Rhizoctonia solani* dsRNA virus 3 (RsRV3) [40,43,48], and RsEV1 (*Rhizoctonia Solani* Endornavirus) have been discovered [49]. *R. solani* isolates frequently have many co-infections; *R. solani* AG2-2 IV DC17, for example, has been reported to carry an endornavirus, a *megabirnavirus*, a *mitovirus*, two *flexiviruses*, and the three invasively related *mycoalphaviruses* [41]. Similarly, *R. solani* AG-3PT RS002, which infects potatoes, is home to both an endornavirus and a mitovirus [50,51].

## 7. dsRNA Mycoviruses

**Figure A2:**
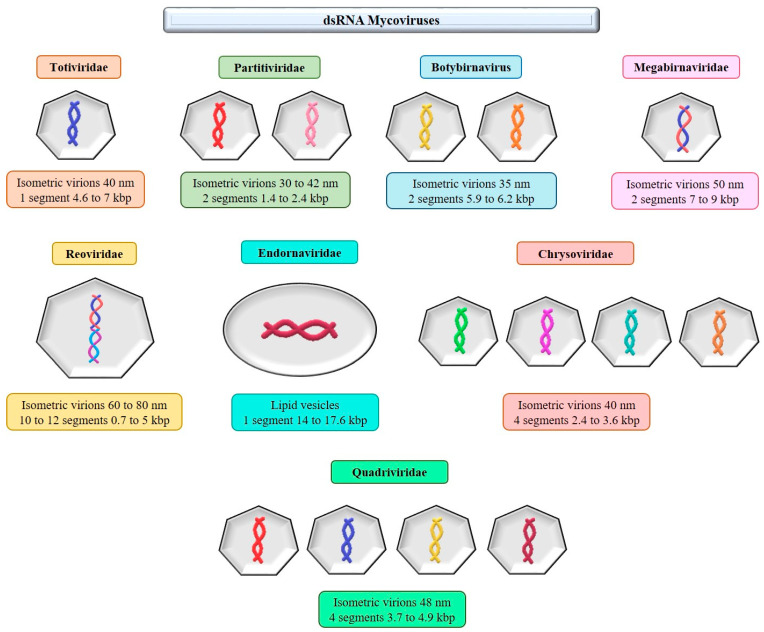
Analysis of dsRNA mycoviruses.

Most mycoviruses with dsRNA genomes are encapsidated in isometric particles [52]. Currently, there are eight families that fall under *Amalgaviridae* (one genomic segment, length 3.5 kbp), *Megabirnaviridae* (two genomic segments, length 7.0–9.0 kbp), *Chrysoviridae* (three to seven genomic segments, length 2.4–3.6 kbp), *Partitiviridae* (two to three genomic segments, length 1.4–2.3 kbp), *Reoviridae* (*Spinareovirinae* subfamily, ten to twelve genomic segments [52]. In addition, the *Botrytis porri* RNA virus 1 (BpRV1), a dsRNA virus from the *Botybirnavirus* genus, has been described [53]. The taxonomy of mycoviruses often evolves when new viruses are discovered. New families, such as *Fusariviridae* (four genomic segments 1.5–3.6 kbp in length) and *Alternaviridae* (one genomic segment 6–7 kbp in length), have also recently been proposed [54]. The majority of positive-sense (+) ssRNA fungal and plant viruses, such as *Alphaflexiviridae*, *Betaflexiviridae*, *Gammaflexiviridae*, *Closteroviridae*, and *Potyviridae*, form spherical but not filamentous virions; however, a novel dsRNA virus from *Colletotrichum camelliae* identified from tea plants in China was discovered [52,55]. However, *Beauveria bassiana* polymycovirus-1 (BbPmV-1) and *Aspergillus fumigatus* tetramycovirus-1 (AfuTmV-1) from the insect pathogen *B. bassiana* and the human pathogen *A. fumigatus,* respectively, have been reported recently (Figure A2) [32]. BbPmV-1 exhibits hypovirulence, which is unusual for mycoviruses, in its host [14].

### 7.1. Family Megabirnaviridae

One genus of the *Megabirnaviridae* family, *Megabirnavirus*, is known to infect fungi [56]; another similar genus, Phlegivirus, has also been proposed [30]. Individuals of the family may accept straight bi-segmented dsRNA genomes that are each between 7 and 8.9 kbp long and have a combined length of 16.1 kbp. The isometric particles that contain the dsRNA genomes are packed [30,57]. For each segment, the only officially recognized species *Rosellinia necatrix* megabirnavirus 1 (RnMV1/W779) prototype has two tandems, non-relating ORFs [58]. *Sclerotinia sclerotiorum* megabirnavirus 1 (SsMBV1) [59], *Rosellinia necatrix* megabirnavirus 2 (RnMBV2) [60], *Pleospora* megabirnavirus 1 (PMBV1) [60], and *Entoleuca* megabirnavirus 1 (EnMBV1) have also been described [61]. Viral transmission happens horizontally through anastomosis or vertically through spores that cause the production of new progeny [57]. Moreover, more dsRNA viruses belonging to the Megabirnaviridae family were found using modern NGS methods [30]. Two linear and independently encapsidated monocistronic dsRNA segments are accommodated in members of the Partitiviridae family, while an extra satellite or damaged dsRNA segment may be present. Each dsRNA segment is one big ORF encoding a putative RNA-dependent RNA polymerase (RdRp) or coat protein (CP) and is between 1.4 and 2.4 kbp in size [62]. *Alphapartitivirus*, *Betapartitivirus*, *Deltapartitivirus*, *Cryspovirus*, and *Gammapartitivirus* are the five genera that comprise the family *Partitiviridae* [62]. Fungal partitiviruses can spread vertically through spores or horizontally through hyphal fusion [63].

### 7.2. Family Partitiviridae

*R. solani* has been confirmed to include members *of alphapartitivirus* and *betapartitivirus* genera. The cause of rice diseases such as sheath blight, *Rhizoctonia solani* AG-1 IA, was shown to be an *alphapartitivirus* known as *Rhizoctonia solani* partitivirus *2 (RsPV2)*. Two segments, each measuring 2020 bps and 1790 bps, are supported by RsPV2/GD-11. *Rhizoctonia solani* virus 717 (RsV717) has two genomic parts 2363 and 2206 kbp in length. In addition, the complete coding sequences of the *R. solani* AG2-2 LP-identified *R. solani* partitiviruses 5 to 7 (RsPV6/BR5, RsPV7/BR6, and RsPV8/BR16) and *R. solani* dsRNA virus 2 (RsDSRV2/A) have been characterized using NGS. RsPV6/BR5 is a member of the genus *Betapartitivirus*, while RsDSRV2/A, RsPV8/BR16, and RsPV7/BR6 RdRps are members of the genus *Alphapartitivirus* [64]. Moreover, a fragment of the *Rhizoctonia solani* partitivirus 1 sequence, isolated from *R. solani* OA-1, has been reported.

## 8. Unclassified dsRNA Mycoviruses

A few investigations have shown that *R. solani*, a pathogenic fungus, is infected by unclassified dsRNA viruses. RsRV1 was the first *Rhizoctonia solani* dsRNA virus identified [46]. Among the unique chromosomal dsRNAs L1 (25 kbp), L2 (23 kbp), and S1 (1.2 kbp), M1 and M2 dsRNAs were discovered in *R. solani* Rhs 1A, and they constitute the first thoroughly documented dsRNA elements in the pathogenic fungus *R. solani* [65]. Two putative ORFs are accommodated on the positive strand of M1, being identical to the previously identified *Rhizoctonia solani* virus 1 (RsV1). Four more ORFs have been discovered on the negative strand [66]. M2 has a single main ORF that encodes an RdRp that is related to the penta-functional AROM polypeptide of the shikimate pathway and has an intense relationship with the mycovirus-like *Rhizoctonia solani* and barely synthesizes five intermediate steps in the (biochemical) shikimate pathway in filamentous yeast and fungi [44].

## 9. ssRNA Mycoviruses

**Figure A3:**
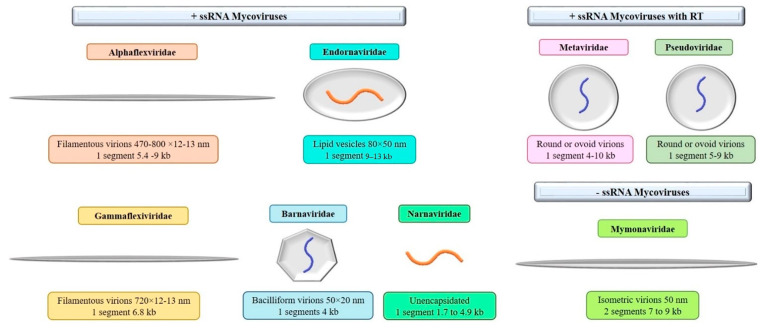
Analysis of ssRNA mycoviruses.

Single-stranded (ss) RNA viruses are also common in *R. solani*, in addition to dsRNA viruses [66]. SsRNA serves as the genetic makeup of the tiniest and most basic viruses [67]. Based on the polarity of their RNA genomes, ssRNA viruses can be categorized as positive-sense (+) or negative-sense (−) RNA. According to Koonin et al. [68], (+) ssRNA viruses have a straightforward RNA replication and expression mechanism, whereas ssRNA viruses start replication by encapsulating their transcription and replication machinery within particles (virions) [69]. The linear monopartite (+) ssRNA genome is present in most known ssRNA fungal viruses [70], including *Alphaflexiviridae* (5.4–9 kbp), *Barnaviridae* (4 kbp), *Botourmiaviridae* (2.9 kbp), *Deltaflexiviridae* (6–8 kbp), *Endornaviridae* (14–17.6 kbp), *Gammaflexiviridae* (6.8 kbp), *Hypoviridae* (9–13 kbp), and *Narnavirid* (1.7–2.9 kbp). *Sclerotinia sclerotiorum* negative-stranded RNA virus 1 has recently been classified as a member of the family *Mymonaviridae* and is the only ssRNA mycovirus the ICTV officially recognizes (Figure A3) [71].

### 9.1. Family Barnaviridae

There is presently just one species and one genus of barnavirus in the family *Barnaviridae* [72]. The model species’ monopartite (+) ssRNA genome, the mushroom bacilliform virus, is 4.0 kbp in length. The whole genome contains four ORFs, each coding a protein with a function (P1) that is unknown, a polyprotein with VPg, and the domains protease (P2), a putative CP (P4), and RdRp (P3); furthermore, the *Rhizoctonia solani* barnavirus 1 (RsBV1) and barnavirus 1, belonging to the genus *barnavirus*, have been identified [73]. The polyprotein encoded by RsBarV1, which has a length of 3915 bp and three ORFs, codes for a protease, the putative domain VPg, a protein that is unknown, a putative CP, and a relationship with MBV. The absence of the ORF, which is thought to encode a hypothetical protein with an unknown function, suggests that the 5′ terminal sequence of RsBV1 is incomplete.

### 9.2. Family Benyviridae

Positive-sense ssRNA viruses of plants with rod-shaped particles (virions) are capped and polyadenylated genomes. Their lengths range from 1.3 to 6.7 kb, and they are found in the family *Benyviridae* [74]. Four species make up the genus *Benyvirus*, and the individuals of this family are linked to cell-to-cell migration [74,75]. *R. solani* 42304-9a and *R. solani* AG-2.2 LP BR2 were discovered to have two different viruses, known as *Rhizoctonia solani Beny*-like virus 1 (RsBenV1) and *Rhizoctonia solani Beny*-like virus 3 (RsBenV3), which were both identified and partially sequenced. Only one region of each genome was identified as encoding a putative RdRp associated with *benyviruses* and beny-like viruses. RsBenV1/42304-9 shares a close resemblance to a recognized individual of the *Benyviridae* family, whereas RsBenV1/BR2 resembles the *Sclerotium rolfsii* beny-like virus 1.

### 9.3. Family Botourmiaviridae

Ten species and four genera make up the family *Botourmiaviridae* (*Botoulivirus* (2.9 kbp long), *Magoulivirus* (2.3 kbp long), *Ourmiavirus* (three segments, 0.9 kbp, 1.0 kbp, and 2.8 kbp long), and *Scleroulivirus* (one segment, 3 kb long)). Just 59% to 87% of the *Rhizoctonia solani* ourmia-like virus 1 (RsOLV1) genome has been described, and an essential investigation revealed similarities to the RdRps of *Ourmiavirus* genus members, including the Cassava virus C, Ourmia melon virus, and the Epirus cherry [73,76]. It is thought that plant viruses of the *Ourmiavirus* genus developed by reassorting the genomic segments of viruses that infected both plants and fungi. These viruses are tripartite, with each segment coding for one protein: RdRp, putative CP, and protein movement (MP) [74]. Nevertheless, the putative CP and MP are not encoded in the RsOLV1 genome [73,76]. According to recent research, the mono-segmented S. sclerotiorum ourmia-like virus 4 (SsOLV4) was isolated from the fungus *S. sclerotiorum* and solely encoded the RdRp protein, which is sufficient for infection, replication, and transmission. Moreover, research on SsOLV4 implies that the family *Botourmiaviridae* needs a new genus. As a result, fungal ourmia-like viruses only have one segment at this time. Today, Rhizoctonia magoulivirus 1 (RsOLV1) is the family *Botourmiaviridae*, a *Magoulivirus* genus and legally recognized species. In addition, the evolutionary phylogenetic tree grouped RsOLV 2–5 and *Agaricus bisporus* virus 15 into a potentially new genus within the family *Botourmiaviridae*, and even shows a new relationship with the family that is named *Basidionarnaviridae* [64]. About 70% similarity exists between the RsOLV2/Rs, RsOLV2, and RsOLV3 RdRp sequences. As a result, they are probably distinct isolates of the same species [64].

### 9.4. Family Bromoviridae

*Alfamovirus*, *Anulavirus*, *Bromovirus*, *Cucumovirus*, *Ilarvirus*, and *Oleavirus* are six of the family’s current genera. The tripartite linear (+) ssRNA genome of the *Bromoviridae* family is around 8 kb long [77,78]. Putative RdRp 1a and 2a are encoded by RNA1 and RNA2. Members of the genera *Cucumovirus* and *Ilarvirus* have an extra overlapping ORF. For the individuals of the genera *Cucumovirus*, *Anulavirus*, *Ilarvirus*, and *Bromovirus*, the family *Bromoviridae* generates particles (virions) that are either spherical or quasi-spherical or bacilliform, like the members of the genera *Alfamovirus*, *Ilarvirus,* and *Oleavirus* [77].

### 9.5. Tymoviridae, Deltaflexiviridae, and Unidentified Viruses of the Tymovirales Order Are Mycoviruses

The families *Alphaflexiviridae*, *Deltaflexiviridae*, *Betaflexiviridae*, and *Gammaflexiviridae* are highlighted by the collective name “*flexiviruses*” which belongs to the order *Tymovirales*. The filamentous virions of flexiviruses possess a monopartite (+) ssRNA polyadenylated genome that is between 6.5 and 9.5 kb long and codes for a replication-associated polyprotein between 150 and 250 kDa in size [70]. It is known that the flexiviruses may infect both fungi and plants [79]. *Botrytis* virus F (BotV-F), which is related to the family *Gammaflexiviridae* and the genus *Mycoflexivirus*, was the first mycovirus identified in the order *Tymovirales* [80]. Three species belonging to the genus *Deltaflexivirus* have been described within the family *Deltaflexiviridae*: *Sclerotinia deltaflexivirus* 1 (SsDFV1), *soybean-associated deltaflexivirus* 1 (SlaMFV1), and *Fusarium deltaflexivirus* 1 (FgDFV1) [70]. *Rhizoctonia solani flexivirus* 1 (RsFV-1) is the only flexivirus that is fully sequenced and has been found to affect the pathogenic fungus *R. solani* [41]. The (+) ssRNA genome of RsFV-1, separate from *R. solani* AG2- 2IV/DC17, has 10,621 nts without the poly-A tail. One protein, encoded by RsFV-1, is connected to those of other *Tymovirales* members [41]. *Tymoviridae* is a family that includes (+) ssRNA viruses between 6.0 and 7.5 kb in size [81]. Forty-one officially identified species are in the family *Tymoviridae*, including three genera: *Marafivirus*, *Maculavirus*, and *Tymovirus* [82]. 

### 9.6. Family Endornaviridae

A family of viruses known as *Endornaviridae* has RNA genomes that are unencapsidated, and their size ranges from 8.7 to 16.6 kb. Just one ORF in the family codes for a polyprotein [83]. The polyprotein has conserved RdRp domain motifs at the C-terminus and an RNA helicase domain at the N-terminus [84]. *Endornaviruses* are chronic infections of plants, fungi, and oomycetes that do not manifest symptoms in their hosts [84,85]. In fungal hosts, members of *Endornaviridae* are transmitted vertically through sporulation and horizontally through anastomosis [86], while in plant hosts, they are spread via vertical transmission through pollen and ova since they lack a putative MP and cannot move from cell to cell. An endornavirus identified from *R. solani* AG-3PT strain RS002 (RsEV-RS002) was partially characterized and tentatively entitled *Rhizoctonia solani* endornavirus.

### 9.7. Family Hypoviridae

One identified species, Hypovirus, and four others with capsidless, mono-segmented (+) ssRNA genomes with a size ranging from 12.7 to 9.2 Kbp make up the family *Hypoviridae* [87]. One or two ORFs each have a genome that encodes the domains RdRp and Hel [88]. Additional domain motifs, such as papain-like protease (PRO), glucosyltransferase (UGT), and permuted papain-fold peptidase of dsRNA viruses and eukaryotes (PPPDE), are also present [89,90]. The capacity of hypoviruses to reduce the fungal host virulence (hypovirulence) of the chestnut blight pathogenic fungus *Cryphonectria* parasitica initially sparked interest in them. Via hyphal anastomosis, the members of the hypovirus can spread to virulent isolates horizontally [90]. To the best of our knowledge, a complete *R. solani* hypovirus genome has not yet been found; nevertheless, the NGS technique was previously used to identify whole ORFs of *Rhizoctonia solani* hypovirus 1 (RsHV1) and partial ORFs of *Rhizoctonia solani* hypoviruses 2 and 3 (RsHV2 and 3, respectively) [64]. With two ORFs each, genomic sequences of RsHV2 and 3 are 9 and 5 kbp long. Whereas one RsHV3 ORFs encodes a helicase-conserved domain motif, both RsHV2 ORFs produce hypovirus-related proteins without any putative conserved features. In the family *Hypoviridae*, a new genus, *Megahypovirus*, was also suggested to fit the huge genomes of RsHV1 and SsHV2, as well as RsHV2, RsHV3, and *Agaricus bisporus* virus 2 [64]. Also, three fusariviruses were found to be homologous to the members of the proposed family, *Fusariviridae*. *Rhizoctonia solani* fusarivirus 1, 2, and 3 (RsFV1, 2, and 3, respectively) were described [91]. The 11 kb long RsFV1 genomic segment contains four putative ORFs. ORF1 encodes a viral helicase. ORF2 and 4 were short ORFs and no significant homology with other proteins. The largest ORF3 codes for a protein with putative RdRp and Hel domains. The genomic architecture of the RsFV2 is identical; however, the incomplete genomic sequence of RsFV3 codes for putative RdRp and Hel domains [64].

### 9.8. Mitoviridae and Narnaviridae Families

The simplest known viruses are those belonging to the *Narnaviridae* and *Mitoviridae* families. Their linear (+) ssRNA genomes, which range in size from 1.7 to 3.6 kb, include a single ORF that encodes a putative RdRp and lacks a capsid. *Mitoviridae* and *Narnaviridae* each have one genus, *Mitovirus* (five species) and *Narnavirus* (two species), respectively [92]. According to Nerva et al. [93], all known members of the genus Mitovirus infect filamentous plants, and a fungal *Narnavirus* has also been discovered [53]. Although mitoviruses and narnaviruses do not produce viral particles, they are linked to lipid-membrane-bound vesicles with genomes between 2.3 and 3.1 kb in length [40,94]. In contrast to members of the narnavirus family, which are known to replicate in the cytosol, mitoviruses reproduce in the host cell’s mitochondria. Since the first Mitovirus was found in *C. parasitica*, many mitoviruses have been found in phytopathogenic fungi [40]. The pathogenic fungus *R. solani* does not include any narnaviruses or complete mitovirus genome sequences. Forty incomplete mitovirus genome sequences associated with *R. solani* have been identified [30,65,95].

### 9.9. Family Togaviridae

The Eastern equine encephalitis virus (EEEV), the Western equine encephalitis virus (WEEV), the Venezuelan equine encephalitis virus (VEEV), the Sindbis virus (SINV), the Ross River virus (RRV), the Semliki Forest virus (SFV), and the Chikun virus (SFV) are all members of the *Togaviridae* family, which includes 31 species in total [96]. Arboviruses called alphaviruses alternately infect vertebrate hosts and are spread by insect vectors [97]. Small enveloped (+) ssRNA viruses with a genome encoding structural and non-structural proteins are found in members of the *Togaviridae* family and range in size from 10 to 2 Kbp [98]. The viral particles comprise surface glycoproteins, a lipid bilayer, and a nucleocapsid core [98]. To the best of our knowledge, *R. solani* is not infected by any members of the genus *Alphavirus.* However, recently, partial genomic sequences belonging to the *Togaviridae* family, including *Rhizoctonia solani* alphavirus-like 1, 2, and 3, were found in *R. solani* AG-2 LPin (RsALV1/BR15, RsALV2/BR14, and RsALV3/BR8) [64]. The RsAVL2 partial ORF encodes a putative RdRp, whereas the RsAVL1 and RsAVL3 partial ORFs include both putative viral helicase and RdRp domains [63].

### 9.10. Order Serpentovirales

*Aspiviridae*, formerly known as *Ophioviridae*, is a family of adaptable filamentous viruses that belong to the order *Serpentovirales* and are known to infect plants [99]. Currently, the family *Aspiviridae* has seven species, including the genus *Ophiovirus.* The (−) ssRNA genomes of the family *Aspirividae* range in size from 11.3 to 12.5 kb and are divided into three to seven segments [99]. *Rhizoctonia solani* negative-stranded RNA virus 1-3 (RsNSRV1-3) is a recently identified ophiovirus-related incomplete viral genome sequence that infects soil-borne *R. solani* strains. The lettuce ring necrosis ophiovirus and other members of the *Aspiviridae* family have RNA1s that encode big ORFs with considerable similarities to the L-protein domain. The *Fusarium poae* negative-stranded RNA virus1 (FpNSV1), isolated from the pathogenic fungus *Fusarium poae*, has also been proposed as a new family, *Betamycoserpentoviridae*, in the order *Serpentovirales* [73].

### 9.11. Order Bunyavirales

The Arenaviridae, Cruliviridae, Hantaviridae, Fimoviridae, Leishbuviridae, Mypoviridae, Nairoviridae, Phasmaviridae, Peribunyaviridae, Phenuiviridae, Tospoviridae, and Wupedeviridae families are considered herein. Many (ssRNA) fungal viruses linked to bi- and tri-segmented (ssRNA) viruses, like phenuiviruses and peribunyaviruses, were found in plant-pathogenic fungi after metatranscriptomics analysis [73]. For instance, the first segmented (ssRNA) virus discovered to infect fungus is the Lentinula edodes negative-stranded RNA virus 2 (LeNSRV2), which also infects Lentinula edodes [45]. In contrast, more viruses belonging to the order Bunyavirales were found in fungi connected to the ascomycete Entoleuca spp. and the marine organism *Holothuria polii* [61].

## 10. Effects of Viral Infection in *Rhizoctonia solani*

**Figure A4:**
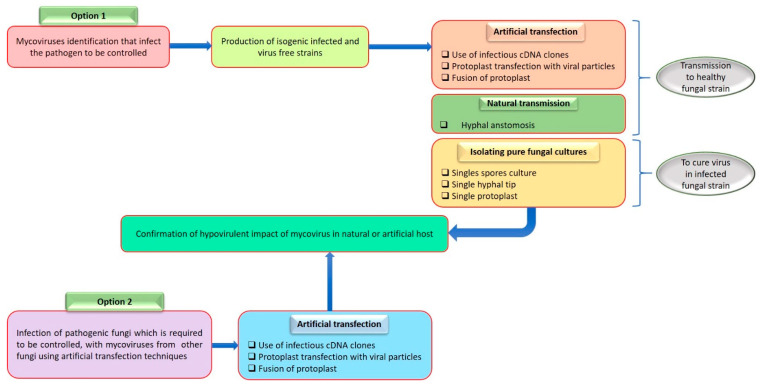
Detection of viruses that reduce virulence of fungal plant pathogens. To demonstrate mycovirus hypovirulence, isogenic infected and virus-free strains were created using various methods. These methods can also be used to transfer viruses to fungal hosts and test heterologous mycovirus control strategies.

Mycovirus infections are frequently asymptomatic (cryptic), although research focuses on possible hypovirulence, a feature that might emerge in the setting of long-term biological control of fungi. The best example is *Cryphonectria hypovirus* 1 (CHV1), which was successfully used in Europe to eradicate the plant disease *Cryphonectria parasitica*, the cause of chestnut blight [35]. The term “hypovirulence” was coined due to this discovery, which completely changed the field of fungus biological management [100]. The “La France” illness of Agaricus biporus caused by the “La France” isometric virus (LIV) and other mycoviruses may also have more deadly consequences. These fungi infections are caused by the OMSV and OMIV (oyster mushroom spherical and isometric viruses) [95]. To study how mycoviruses affect their host, it is essential to create an isogenic line that is virus-free, either by curing the virus-infected individual or transferring the mycovirus into a virus-free strain (Figure A4) [32].

## 11. Conclusions

One of the most significant fungal diseases of rice in the world is rice sheath blight. One of the primary issues limiting the growth of ratoon rice is the risk of the occurrence of rice sheath blight, which prevents the axillary bud germination of ratoon rice. Due to *R. solani’s* wide host range and soil-borne and saprotrophic nature, attempts to manage rice sheath blight using agronomic measures, such as breeding techniques, chemical fungicides, and crop rotation, have been ineffectual. The pathogenic fungus *R. solani* showed tenacity even when large amounts of chemicals, such as fungicides, were used because of significant crop losses. The fungus *R. solani* is notable for not producing conidia (asexual spores), so its capacity to be widely distributed in soil is limited. Another review on these viruses is being carried out to identify pathogenicity-related hypovirulence from *R. solani* while supplying novel viral resources and techniques for the biological control of *R. solani*-caused illness. The review will present advanced knowledge of fungal virus varieties, evolution, interaction with hosts, and the application of fungal viruses in managing sheath blight disease. This review will advance our understanding of the evolution of the many mycoviruses. Also, this review will increase our understanding of new mycoviruses and their potential application as biological control agents against plant-pathogenic fungi.

## 12. Future Aspects

To safeguard crops like rice (*O. sativa*) from *R. solani* while minimizing the use of chemical fungicides, new options must be developed, preferably with minimal environmental effects. Using fungal viruses to manage agricultural diseases has advanced recently. Moreover, it is known that some viruses, some of which are currently unidentified, are hosted by the pathogenic fungus *R. solani*. Mycoviruses from *R. solani* primarily linked to hypovirulence or potentially acting as biocontrol agents can be screened to learn new information and develop a new preventative technology system for managing *R. solani.*

## Data Availability

Data sharing does not apply to this article.

## Appendix A

**Table A1:** The approved mycovirus classifications (released by ICTV 2018b).

Genome	Family/Sub-Family	Genus	Species
ssDNA	*Genomoviridae*	*Gemycircularvirus*	*Sclerotinia gemycircularvirus 1*
dsRNA	*Amalgaviridae*	*Zybavirus*	*Zygosaccharomyces bailii virus Z*
		*Alphachrysovirus*	*Penicillium chrysogenum virus*
	*Chrysoviridae*	*Betachrysovirus*	*Botryosphaeria dothidea chrysovirus*
	*Megabirnaviridae*	*Megabirnavirus*	*Rosellinia necatrix megabirnavirus 1*
		*Alphapartitivirus*	*Rosellinia necatrix partitivirus 2*
		*Betapartitivirus*	*Fusarium poae virus 1*
	*Partitiviridae*	*Cryspovirus*	*Cryptosporidium parvum virus 1*
		*Gammapartitivirus*	*Penicillium stoloniferum virus S*
	*Quadriviridae*	*Quadrivirus*	*Rosellinia necatrix quadrivirus 1*
	*Reoviridae/Spinareovirinae*	*Mycoreovirus*	*Mycoreovirus 1*
		*Totivirus*	*Saccharomyces cerevisiae virus L-A*
	*Totiviridae*	*Victorivirus*	*Magnaporthe oryzae virus 1*
	*Unclassified*	*Botybirnavirus*	*Botrytis porri botybirnavirus 1*
(+) ssRNA	*Alphaflexiviridae*	*Botrexvirus*	*Botrytis virus X*
		*Sclerodarnavirus*	*Sclerotinia sclerotiorum debilitation-associated RNA virus*
	*Barnaviridae*	*Barnavirus*	*Mushroom bacilliform virus*
	*Botourmiaviridae*	*Botoulivirus*	*Botrytis botoulivirus*
		*Magoulivirus*	*Magnaporthe magoulivirus*
		*Scleroulivirus*	*Sclerotinia scleroulivirus 1*
	*Deltaflexiviridae*	*Deltaflexivirus*	*Sclerotinia deltaflexivirus 1*
	*Endornaviridae*	*Alphaendornavirus*	*Oryza sativa alphaendornavirus*
		*Betaendornavirus*	*Sclerotinia sclerotiorum betaendornavirus 1*
	*Gammaflexiviridae*	*Mycoflexivirus*	*Botrytis virus F*
	*Hypoviridae*	*Hypovirus*	*Cryphonectria hypovirus 1*
	*Narnaviridae*	*Mitovirus*	*Cryphonectria mitovirus 1*
		*Narnavirus*	*Saccharomyces 20S RNA narnavirus*
(−) ssRNA	*Mymonaviridae*	*Sclerotimonavirus*	*Sclerotinia sclerotimonavirus*
ssRNA-RT	*Metaviridae*	*Metavirus*	*Saccharomyces cerevisiae Ty3 virus*
	*Pseudoviridae*	*Hemivirus*	*Saccharomyces cerevisiae Ty5 virus*
		*Pseudovirus*	*Saccharomyces cerevisiae Ty1 virus*

## References

[1] Srivastava S., Bist V., Srivastava S., Singh P.C., Trivedi P.K., Asif M.H., Chauhan P.S., Nautiyal C.S. (2016). Unraveling Aspects of *Bacillus amyloliquefaciens* Mediated Enhanced Production of Rice under Biotic Stress of *Rhizoctonia solani*. Front. Plant Sci..

[2] Ge S., Sang T., Lu B.R., Hong D.Y. (1999). Phylogeny of Rice Genomes with Emphasis on Origins of Allotetraploid Species. Proc. Natl. Acad. Sci. USA.

[3] Sanchez P.L., Wing R.A., Brar D.S., Zhang Q., Wing R.A. (2013). The Wild Relative of Rice: Genomes and Genomics. Genetics and Genomics of Rice.

[4] Vaughan D.A. (1994). The Wild Relatives of Rice: A Genetic Resources Handbook.

[5] Sreenivasaprasad S., Johnson R., Banniza S., Holderness M. (2001). Rice Sheath Blight—Pathogen Biology and Diversity. Major Fungal Diseases of Rice.

[6] Padasht-Dehkaei F., Ceresini P.C., Zala M., Okhovvat S.M., Nikkhah M.J., McDonald B.A. (2012). Population genetic evidence that basidiospores play an important role in the disease cycle of rice-infecting populations of *Rhizoctonia solani* AG-1 IA in Iran. Plant Pathol..

[7] Lee F.N. (1983). Rice Sheath Blight: A Major Rice Disease. Plant Dis..

[8] Xia Y., Fei B., He J., Zhou M., Zhang D., Pan L., Li S., Liang Y., Wang L., Zhu J. (2017). Transcriptome Analysis Reveals the Host Selection Fitness Mechanisms of the *Rhizoctonia solani* Ag1ia Pathogen. Sci. Rep..

[9] Gunnell P.S., Webster R.K. (1984). Aggregate sheath spot of rice in California. Plant Dis..

[10] Johanson A. (1998). A PCR-Based Method to Distinguish Fungi of the Rice Sheath-Blight Complex, *Rhizoctonia solani*, *R. oryzae* and *R. oryzae*-*sativae*. FEMS Microbiol. Lett..

[11] Andika I.B., Wei S., Cao C., Salaipeth L., Kondo H., Sun L. (2017). Phytopathogenic Fungus Hosts a Plant Virus: A Naturally Occurring Cross-Kingdom Viral Infection. Proc. Natl. Acad. Sci. USA.

[12] Abdoulaye A.H., Foda M.F., Kotta-Loizou I. (2019). Viruses Infecting the Plant Pathogenic Fungus *Rhizoctonia solani*. Viruses.

[13] Slaton N.A., Cartwright R.D., Meng J., Gbur E.E., Norman R.J. (2003). Sheath Blight Severity and Rice Yield as Affected by Nitrogen Fertilizer Rate, Application Method, and Fungicide. Agron. J..

[14] Xie J., Jiang D. (2014). New Insights into Mycoviruses and Exploration for the Biological Control of Crop Fungal Diseases. Annu. Rev. Phytopathol..

[15] Gobbin D., Hoegger P.J., Heiniger U., Rigling D. (2003). Sequence Variation and Evolution of *Cryphonectria hypovirus* 1 (CHV-1) in Europe. Virus Res..

[16] Umer M., Qadeer A., Razaq Z., Anwar N., Kiptoo J.J. (2022). Mycovirus: Biocontrol Agent against *S. sclerotiorum* of Rapeseed. Phytopathogenom. Dis. Control.

[17] Ram R.M., Singh H. (2018). *Rhizoctonia bataticola*: A Serious Threat to Chickpea Production. Int. J. Chem. Stud..

[18] Anderson J.P., Kidd B.N., Garg G., Singh K.B. (2018). Transcriptome Analysis Reveals Class IX Ethylene Response Factors Show Specific Up-Regulation in Resistant but Not Susceptible *Medicago truncatula* Lines Following Infection with *Rhizoctonia solani*. Eur. J. Plant Pathol..

[19] Chi Y., Xu M., Yang J., Wang F., Wu J. (2016). First Report of *Rhizoctonia solani* Causing Peanut Pod Rot in China. Plant Dis..

[20] Kiptoo J.J., Abbas A., Bhatti A.M., Usman H.M., Shad M.A., Umer M., Atiq M.N., Alam S.M., Ateeq M., Khan M. (2021). *Rhizoctonia solani* of Potato and its Management: A Review. Plant Protect..

[21] Agrios G. (2005). Plant Pathology.

[22] Uppala S., Zhou X. (2018). Rice Sheath Blight. Plant Health Instr..

[23] Ghosh P., Sen S., Chakraborty J., Das S. (2016). Monitoring the Efficacy of Mutated *Allium sativum* Leaf Lectin in Transgenic Rice against *Rhizoctonia solani*. BMC Biotechnol..

[24] Costanzo S., Jackson A.K., Brooks S.A. (2011). High-resolution Mapping of Rsn1, A Locus Controlling Sensitivity of Rice to a Necrosis-Inducing Phytotoxin from *Rhizoctonia solani* AG1-IA. Theor. Appl. Genet..

[25] Chen Y., Yao J., Yang X., Zhang A.-F., Gao T.-C. (2014). Sensitivity of *Rhizoctonia solani* Causing Rice Sheath Blight to Fuxapyroxad in China. Eur. J. Plant Pathol..

[26] de França S.K.S., Cardoso A.F., Lustosa D.C., Ramos E.M.L.S., de Filippi M.C.C., da Silva G.B. (2014). Biocontrol of Sheath Blight by *Trichoderma asperellum* in Tropical Lowland Rice. Agron. Sustain. Dev..

[27] Son M., Yu J., Kim K.-H. (2015). Five Questions about Mycoviruses. PLoS Pathog..

[28] Shafik K., Umer M., You H., Aboushedida H., Wang Z., Ni D., Xu W. (2021). Characterization of a Novel Mitovirus Infecting *Melanconiella theae* Isolated from Tea Plants. Front. Microbiol..

[29] Li Z., Chen L., Meiling Z., Mei Y., Erxun Z. (2018). Diversity of DsRNA Viruses Infecting Rice Sheath Blight Fungus *Rhizoctonia solani* AG-1 IA. Rice Sci..

[30] Bartholomäus A., Wibberg D., Winkler A., Pühler A., Schlüter A., Varrelmann M. (2016). Deep Sequencing Analysis Reveals the Mycoviral Diversity of the Virome of an Avirulent Isolate of *Rhizoctonia solani* AG-2-2 IV. PLoS ONE.

[31] Abbas A. (2016). A Review Paper on Mycoviruses. J. Plant Pathol. Microbiol..

[32] Kotta-Loizou I., Coutts R.H.A. (2017). Mycoviruses in Aspergilli: A Comprehensive Review. Front. Microbiol..

[33] Hillman B.I., Annisa A., Suzuki N. (2018). Viruses of Plant-Interacting Fungi. Adv. Virus Res..

[34] Eusebio-Cope A., Sun L., Tanaka T., Chiba S., Kasahara S., Suzuki N. (2015). The Chestnut Blight Fungus for Studies on Virus/Host and Virus/Virus Interactions: From a Natural to a Model Host. Virology.

[35] Feau N., Dutech C., Brusini J., Rigling D., Robin C. (2014). Multiple Introductions and Recombination in *Cryphonectria hypovirus* 1: Perspective for a Sustainable Biological Control of Chestnut Blight. Evol. Appl..

[36] Brusini J., Robin C. (2013). Mycovirus Transmission Revisited by in Situ Pairings of Vegetatively Incompatible Isolates of *Cryphonectria parasitica*. J. Virol. Methods.

[37] Zilio G., Thiévent K., Koella J.C. (2018). Host Genotype and Environment Affect the Trade-off between Horizontal and Vertical Transmission of the Parasite *Edhazardia aedis*. BMC Evol. Biol..

[38] Frank A., Saldierna Guzmán J., Shay J. (2017). Transmission of Bacterial Endophytes. Microorganisms.

[39] Ghabrial S.A., Castón J.R., Jiang D., Nibert M.L., Suzuki N. (2015). 50-plus Years of Fungal Viruses. Virology.

[40] Liu J.-J., Chan D., Xiang Y., Williams H., Li X.-R., Sniezko R.A., Sturrock R.N. (2016). Characterization of Five Novel Mitoviruses in the White Pine Blister Rust Fungus Cronartium Ribicola. PLoS ONE.

[41] Zheng L., Zhang M., Chen Q., Zhu M., Zhou E. (2014). A Novel Mycovirus Closely Related to Viruses in the Genus Alphapartitivirus Confers Hypovirulence in the Phytopathogenic Fungus *Rhizoctonia solani*. Virology.

[42] Jian J., Lakshman D.K., Tavantzis S.M. (1997). Association of Distinct Double-Stranded RNAs with Enhanced or Diminished Virulence in *Rhizoctonia solani* Infecting Potato. Mol. Plant Microbe Interact..

[43] Chen Y., Tong Gai X., Xing Chen R., Li C.X., Zhao G.K., Yuan Xia Z., Zou C.M., Zhong J. (2019). Characterization of Three Novel Betapartitiviruses Co-Infecting the Phytopathogenic Fungus *Rhizoctonia solani*. Virus Res..

[44] Lakshman D.K., Jian J., Tavantzis S.M. (1998). A Double-Stranded RNA Element from a Hypovirulent Strain of *Rhizoctonia solani* Occurs in DNA Form and Is Genetically Related to the Pentafunctional AROM Protein of the Shikimate Pathway. Proc. Natl. Acad. Sci. USA.

[45] Lin Y.-H., Fujita M., Chiba S., Hyodo K., Andika I.B., Suzuki N., Kondo H. (2019). Two Novel Fungal Negative-Strand RNA Viruses Related to Mymonaviruses and Phenuiviruses in the Shiitake Mushroom (*Lentinula edodes*). Virology.

[46] Zheng L., Liu H., Zhang M., Cao X., Zhou E. (2013). The Complete Genomic Sequence of a Novel Mycovirus from *Rhizoctonia solani* AG-1 IA Strain B275. Arch. Virol..

[47] Zhong J., Chen C.-Y., Gao B.-D. (2015). Genome Sequence of a Novel Mycovirus of *Rhizoctonia solani*, a Plant Pathogenic Fungus. Virus Genes.

[48] Zhang M., Zheng L., Liu C., Shu C., Zhou E. (2018). Characterization of a Novel DsRNA Mycovirus Isolated from Strain A105 of *Rhizoctonia solani* AG-1 IA. Arch. Virol..

[49] Zheng L., Shu C., Zhang M., Yang M., Zhou E. (2019). Molecular characterization of a novel endornavirus conferring hypovirulence in rice sheath blight fungus *Rhizoctonia solani* AG- 1 IA Strain GD-2. Viruses.

[50] Das S., Falloon R.E., Stewart A., Pitman A.R. (2014). Molecular Characterization of an Endornavirus from *Rhizoctonia solani* AG-3PT Infecting Potato. Fungal Biol..

[51] Das S., Falloon R.E., Stewart A., Pitman A.R. (2016). Novel Mitoviruses in *Rhizoctonia solani* AG- 3PT Infecting Potato. Fungal Biol..

[52] Jia H., Dong K., Zhou L., Wang G., Hong N., Jiang D., Xu W. (2017). A DsRNA Virus with Filamentous Viral Particles. Nat. Commun..

[53] Wu M., Zhang J.F., Yang J., Jiang L., Li D. (2012). Characterization of a Novel Bipartite Double- Stranded RNA Mycovirus Conferring Hypovirulence in the Phytopathogenic Fungus *Botrytis porri*. J. Virol..

[54] Zhai L., Zhang M., Hong N., Xiao F., Fu M., Xiang J., Wang G. (2018). Identification and Characterization of a Novel Hepta-Segmented DsRNA Virus from the Phytopathogenic Fungus *Colletotrichum fructicola*. Front. Microbiol..

[55] Lau S.K.P., Lo G.C.S., Chow F.W.N., Fan R.Y.Y., Cai J.J., Yuen K.-Y., Woo P.C.Y. (2018). Novel Partitivirus Enhances Virulence of and Causes Aberrant Gene Expression in *Talaromyces marneffei*. MBio.

[56] Sato Y., Miyazaki N., Kanematsu S., Xie J., Ghabrial S.A., Hillman B.I., Suzuki N., Ictv Report Consortium (2019). ICTV Virus Taxonomy Profile: Megabirnaviridae. J. Gen. Virol..

[57] Chiba S., Salaipeth L., Lin Y.-H., Sasaki A., Kanematsu S., Suzuki N. (2009). A Novel Bipartite Double- Stranded RNA Mycovirus from the White Root Rot Fungus *Rosellinia necatrix*: Molecular and Biological Characterization, Taxonomic Considerations, and Potential for Biological Control. J. Virol..

[58] Wang M., Wang Y., Sun X., Cheng J., Fu Y., Liu H., Jiang D., Ghabrial S.A., Xie J. (2015). Characterization of a Novel Megabirnavirus from Sclerotinia Sclerotiorum Reveals Horizontal Gene Transfer from Single-Stranded RNA Virus to Double-Stranded RNA Virus. J. Virol..

[59] Sasaki A., Nakamura H., Suzuki N., Kanematsu S. (2016). Characterization of a New Megabirnavirus That Confers Hypovirulence with the Aid of a Co-Infecting Partitivirus to the Host Fungus, *Rosellinia necatrix*. Virus Res..

[60] Nerva L., Ciuffo M., Vallino M., Margaria P., Varese G.C., Gnavi G., Turina M. (2016). Multiple Approaches for the Detection and Characterization of Viral and Plasmid Symbionts from a Collection of Marine Fungi. Virus Res..

[61] Velasco L., Arjona-Girona I., Cretazzo E., López-Herrera C. (2019). Viromes in Xylariaceae Fungi Infecting Avocado in Spain. Virology.

[62] Vainio E.J., Chiba S., Ghabrial S.A., Maiss E., Roossinck M., Sabanadzovic S., Suzuki N., Xie J., Nibert M., Ictv Report Consortium (2018). ICTV Virus Taxonomy Profile: Partitiviridae. J. Gen. Virol..

[63] Xiao X., Cheng J., Tang J., Fu Y., Jiang D., Baker T.S., Ghabrial S.A., Xie J. (2014). A Novel Partitivirus That Confers Hypovirulence on Plant Pathogenic Fungi. J. Virol..

[64] Picarelli M.A.S.C., Forgia M., Rivas E.B., Nerva L., Chiapello M., Turina M., Colariccio A. (2019). Extreme Diversity of Mycoviruses Present in Isolates of *Rhizoctonia solani* AG2-2 LP from *Zoysia japonica* from Brazil. Front. Cell. Infect. Microbiol..

[65] Jian J., Lakshman D.K., Tavantzis S.M. (1998). A Virulence-Associated, 6.4-Kb, Double-Stranded RNA from *Rhizoctonia solani* Is Phylogenetically Related to Plant Bromoviruses and Electron Transport Enzymes. Mol. Plant Microbe Interact..

[66] Zoll J., Verweij P.E., Melchers W.J.G. (2018). Discovery and Characterization of Novel Aspergillus Fumigatus Mycoviruses. PLoS ONE.

[67] Usui K., Ichihashi N., Yomo T. (2015). A Design Principle for a Single-Stranded RNA Genome That Replicates with Less Double-Strand Formation. Nucleic Acids Res..

[68] Koonin E.V., Dolja V.V. (1993). Evolution and Taxonomy of Positive-Strand RNA Viruses: Implications of Comparative Analysis of Amino Acid Sequences. Crit. Rev. Biochem. Mol. Biol..

[69] Reguera J., Gerlach P., Cusack S. (2016). Towards a Structural Understanding of RNA Synthesis by Negative Strand RNA Viral Polymerases. Curr. Opin. Struct. Biol..

[70] Chen X., He H., Yang X., Zeng H., Qiu D., Guo L. (2016). The Complete Genome Sequence of a Novel *Fusarium graminearum* RNA Virus in a New Proposed Family within the Order *Tymovirales*. Arch. Virol..

[71] Liu L., Xie J., Cheng J., Fu Y., Li G., Yi X., Jiang D. (2014). Fungal Negative-Stranded RNA Virus That Is Related to Bornaviruses and Nyaviruses. Proc. Natl. Acad. Sci. USA.

[72] Revill P.A., Davidson A.D., Wright P.J. (1994). The Nucleotide Sequence and Genome Organization of Mushroom Bacilliform Virus: A Single-Stranded RNA Virus of *Agaricus bisporus* (Lange) Imbach. Virology.

[73] Marzano S.-Y.L., Domier L.L. (2016). Novel Mycoviruses Discovered from Metatranscriptomics Survey of Soybean Phyllosphere Phytobiomes. Virus Res..

[74] Gilmer D., Ratti C., Ictv Report Consortium (2017). ICTV Virus Taxonomy Profile: Benyviridae. J. Gen. Virol..

[75] Kondo H., Hirano S., Chiba S., Andika I.B., Hirai M., Maeda T., Tamada T. (2013). Characterization of Burdock Mottle Virus, a Novel Member of the Genus Benyvirus, and the Identification of Benyvirus-Related Sequences in the Plant and Insect Genomes. Virus Res..

[76] Marzano S.-Y.L., Nelson B.D., Ajayi-Oyetunde O., Bradley C.A., Hughes T.J., Hartman G.L., Eastburn D.M., Domier L.L. (2016). Identification of Diverse Mycoviruses through Metatranscriptomics Characterization of the Viromes of Five Major Fungal Plant Pathogens. J. Virol..

[77] Sztuba-Solinska J., Bujarski J.J. (2008). Insights into the Single-Cell Reproduction Cycle of Members of the Family Bromoviridae: Lessons from the Use of Protoplast Systems. J. Virol..

[78] King A.M., Lefkowitz E., Adams M.J., Carstens E.B. (2011). Virus Taxonomy: Ninth Report of the International Committee on Taxonomy of Viruses.

[79] Ghanem-Sabanadzovic N.A., Tzanetakis I.E., Sabanadzovic S. (2013). Rubus Canadensis Virus 1, a Novel Betaflexivirus Identified in Blackberry. Arch. Virol..

[80] Howitt R.L., Beever R.E., Pearson M.N., Forster R.L. (2001). Genome Characterization of Botrytis Virus F, a Flexuous Rod-Shaped Mycovirus Resembling Plant ‘Potex-like’ Viruses. J. Gen. Virol..

[81] Charles J., Tangudu C.S., Hurt S.L., Tumescheit C., Firth A.E., Garcia-Rejon J.E., Machain-Williams C., Blitvich B.J. (2019). Discovery of a Novel Tymoviridae-like Virus in Mosquitoes from Mexico. Arch. Virol..

[82] Davison A.J. (2017). Journal of General Virology-Introduction to ‘ICTV Virus Taxonomy Profiles. J. Gen. Virol..

[83] Roossinck M.J., Sabanadzovic S., Okada R., Valverde R.A. (2011). The Remarkable Evolutionary History of Endornaviruses. J. Gen. Virol..

[84] Okada R., Kiyota E., Moriyama H., Fukuhara T., Valverde R.A. (2017). Molecular and Biological Properties of an Endornavirus Infecting Winged Bean (*Psophocarpus tetragonolobus*). Virus Genes.

[85] Okada R., Kiyota E., Moriyama H., Toshiyuki F., Valverde R.A. (2014). A New Endornavirus Species Infecting Malabar Spinach (*Basella alba* L.). Arch. Virol..

[86] Okada R., Yong C.K., Valverde R.A., Sabanadzovic S., Aoki N., Hotate S., Kiyota E., Moriyama H., Fukuhara T. (2013). Molecular Characterization of Two Evolutionarily Distinct Endornaviruses Co-Infecting Common Bean (*Phaseolus vulgaris*). J. Gen. Virol..

[87] Hillman B.I., Halpern B.T., Brown M.P. (1994). A Viral dsRNA Element of the Chestnut Blight Fungus with a Distinct Genetic Organization. Virology.

[88] Linder-Basso D., Dynek J.N., Hillman B.I. (2005). Genome Analysis of *Cryphonectria hypovirus* 4, the Most Common Hypovirus Species in North America. Virology.

[89] Aulia A., Andika I.B., Kondo H., Hillman B.I., Suzuki N. (2019). A Symptomless Hypovirus, CHV4, Facilitates Stable Infection of the Chestnut Blight Fungus by a Coinfecting Reovirus Likely through Suppression of Antiviral RNA Silencing. Virology.

[90] Xie J., Xiao X., Fu Y., Liu H., Cheng J., Ghabrial S.A., Li G., Jiang D. (2011). A Novel Mycovirus Closely Related to Hypoviruses That Infects the Plant Pathogenic Fungus *Sclerotinia sclerotiorum*. Virology.

[91] Zhang R., Liu S., Chiba S., Kondo H., Kanematsu S., Suzuki N. (2014). A Novel Single-Stranded RNA Virus Isolated from a Phytopathogenic Filamentous Fungus, *Rosellinia necatrix*, with Similarity to Hypo-like Viruses. Front. Microbiol..

[92] Niu Y., Yuan Y., Mao J., Yang Z., Cao Q., Zhang T., Wang S., Liu D. (2018). Characterization of Two Novel Mycoviruses from *Penicillium digitatum* and the Related Fungicide Resistance Analysis. Sci. Rep..

[93] Nerva L., Forgia M., Ciuffo M., Chitarra W., Chiapello M., Vallino M., Varese G.C., Turina M. (2019). The Mycovirome of a Fungal Collection from the Sea Cucumber *Holothuria polii*. Virus Res..

[94] Marais A., Nivault A., Faure C., Theil S., Comont G., Candresse T., Corio-Costet M.-F. (2017). Determination of the Complete Genomic Sequence of *Neofusicoccum luteum* Mitovirus 1 (NLMV1), a Novel Mitovirus Associated with a Phytopathogenic *Botryosphaeriaceae*. Arch. Virol..

[95] Das S. (2013). *Rhizoctonia solani* on Potato in New Zealand: Pathogen Characterization and Identification of Double-Stranded RNA Viruses That May Affect Their Virulence. Ph.D. Thesis.

[96] Khan A.H., Morita K., Parquet M.D.C., Hasebe F., Mathenge E.G.M., Igarashi A. (2002). Complete Nucleotide Sequence of Chikungunya Virus and Evidence for an Internal Polyadenylation Site. J. Gen. Virol..

[97] Chen R., Mukhopadhyay S., Merits A., Bolling B., Nasar F., Coffey L.L., Powers A., Weaver S.C., Ictv Report Consortium (2018). ICTV Virus Taxonomy Profile: Togaviridae. J. Gen. Virol..

[98] Li L., Jose J., Xiang Y., Kuhn R.J., Rossmann M.G. (2010). Structural Changes of Envelope Proteins during Alphavirus Fusion. Nature.

[99] García M.L., Bó D., Da Graça E., Gago-Zachert J.V., Hammond S., Moreno J., Natsuaki P., Pallás T., Navarro V., Reyes J.A. (2017). ICTV Virus Taxonomy Profile: Ophioviridae. J. Gen. Virol..

[100] Dawe A.L., Nuss D.L. (2013). Hypovirus Molecular Biology: From Koch’s Postulates to Host Self- Recognition Genes That Restrict Virus Transmission. Adv. Virus Res..

